# Yippee like 4 (*Ypel4*) is essential for normal mouse red blood cell membrane integrity

**DOI:** 10.1038/s41598-021-95291-1

**Published:** 2021-08-05

**Authors:** Alexander Mattebo, Taha Sen, Maria Jassinskaja, Kristýna Pimková, Isabel Prieto González-Albo, Abdul Ghani Alattar, Ramprasad Ramakrishnan, Stefan Lang, Marcus Järås, Jenny Hansson, Shamit Soneji, Sofie Singbrant, Emile van den Akker, Johan Flygare

**Affiliations:** 1 Division of Molecular Medicine and Gene Therapy, Lund Stem Cell Center, Lund University, Lund, Sweden; 2 Division of Molecular Hematology, Lund Stem Cell Center, Lund University, Lund, Sweden; 3 Division of Clinical Genetics, Faculty of Medicine, Lund University, Lund, Sweden; 4 Sanquin Research, Department of Hematopoiesis and Landsteiner Laboratory, Amsterdam UMC, University of Amsterdam, Amsterdam, The Netherlands

**Keywords:** Erythropoiesis, Haematopoietic stem cells

## Abstract

The YPEL family genes are highly conserved across a diverse range of eukaryotic organisms and thus potentially involved in essential cellular processes. *Ypel4*, one of five YPEL family gene orthologs in mouse and human, is highly and specifically expressed in late terminal erythroid differentiation (TED). In this study, we investigated the role of *Ypel4* in murine erythropoiesis, providing for the first time an in-depth description of a *Ypel4*-null phenotype in vivo. We demonstrated that the *Ypel4*-null mice displayed a secondary polycythemia with macro- and reticulocytosis. While lack of *Ypel4* did not affect steady-state TED in the bone marrow or spleen, the anemia-recovering capacity of *Ypel4*-null cells was diminished. Furthermore, *Ypel4*-null red blood cells (RBC) were cleared from the circulation at an increased rate, demonstrating an intrinsic defect of RBCs. Scanning electron micrographs revealed an ovalocytic morphology of *Ypel4*-null RBCs and functional testing confirmed reduced deformability. Even though Band 3 protein levels were shown to be reduced in *Ypel4*-null RBC membranes, we could not find support for a physical interaction between YPEL4 and the Band 3 protein. In conclusion, our findings provide crucial insights into the role of *Ypel4* in preserving normal red cell membrane integrity.

## Introduction

The YPEL (Yippee like) family genes were discovered in 2001 when orthologs to the *Drosophila Yippee* gene were identified in a diverse range of eukaryotic organisms^1,2^. Interaction experiments, cloning and sequence analysis of *Yippee* revealed a putative zinc binding RING finger protein with self-interacting properties^1^. The earliest designated human gene ortholog *YPEL1*, “Yippee like 1”, was in comparative analysis found to have four paralogs in the human genome, subsequently named *YPEL2-5*. Mouse gene orthologs (*Ypel1* through *Ypel5*) were also identified with 99.2–100% similarity. Further analysis identified 100 YPEL family genes from 68 species, including mammal, bird, fish, insect and fungus. High homology was detected in all species and a consensus sequence was designated as the Yippee domain^3^. Several studies have implicated localization of the YPEL family proteins to nuclear and perinuclear structures such as the centrosome and mitotic spindle, but also to microtubules^3–5^. Functionally, a common molecular function has not been demonstrated, but studies using reverse genetics approaches have shown effects on proliferation, apoptosis, senescence, cell adhesion and migration^2,5–9^. Most published studies to date have been performed in vitro with cell lines, thereby potentially missing relevant physiological features crucial to the understanding of a common molecular function.

The *Ypel4/YPEL4* gene has been reported to be highly upregulated during terminal erythroid differentiation (TED) in both murine and human erythropoiesis^10–12^, reaching exceptionally high expression levels when compared to other tissues^13^. Erythropoiesis is a tightly regulated process in which red blood cells (RBC) are produced from hematopoietic stem cells (HSC), through a multistep cellular differentiation program involving several intermediate progenitor cells^14,15^. Briefly, rapidly dividing erythroid-restricted progenitors^16–18^ mature, become erythropoietin (EPO)-dependent and upregulate the transferrin receptor CD71. This maturation step initiates TED by synchronization of cell cycle to differentiation, in which each division generates two daughter cells with distinct morphologies, transcriptomes and proteomes^11,12,19,20^. The locking of cell cycle to terminal differentiation allows for timely expression of erythroid genes in a chronological manner and explains the stepwise acquisition of RBC characteristics such as reduction of cell size, hemoglobinization, synthesis and assembly of red cell membrane components as well as end stage enucleation^21^.

Two decades into YPEL family gene-related research, more studies are still needed to highlight the roles of these genes in different biological processes. *Ypel4* has been shown to be a promising gene to study in murine erythropoiesis. Due to this fact and the complex nature of RBC physiology, we hypothesized that *Ypel4* loss-of-function in vivo would generate a phenotype that could elucidate the role of *Ypel4* in RBC-related cellular processes. To evaluate this assumption, we utilized the advantages of reverse genetics techniques and acquired a conventional knockout mouse model^22^. We showed that *Ypel4-*null mice displayed a secondary polycythemia due to an increased clearance of circulating RBCs. Furthermore, *Ypel4*-null RBCs had an ovalocytic morphology and reduced deformability. Band 3 protein levels were slightly reduced in RBC membranes, providing support for a mild red cell membrane disorder in the *Ypel4*-null mice. In conclusion, we for the first time identify *Ypel4* as a regulator of RBC morphology and red cell membrane protein composition.

## Results

### Disruption of the *Ypel4* gene results in a macrocytic secondary polycythemia

**Figure 1:**
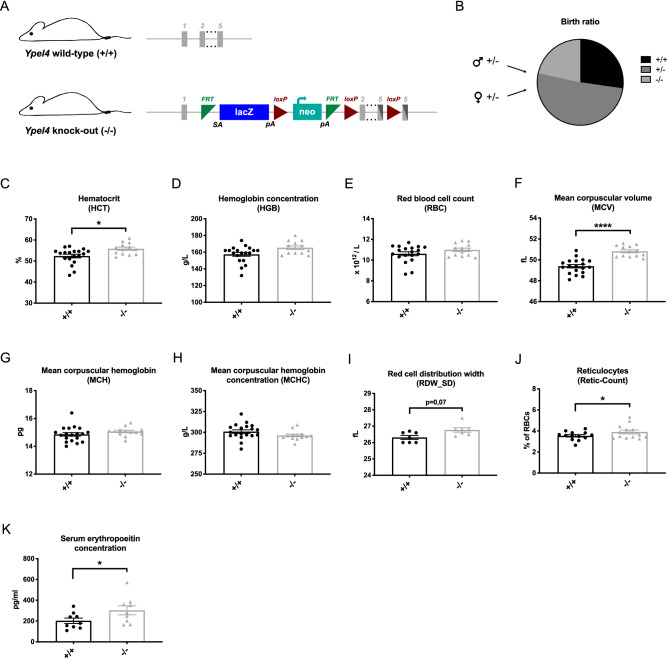
Disruption of the *Ypel4* gene results in a macrocytic secondary polycythemia. (**A**) Schematic of the *Ypel4* wild-type (+) and knockout (−) allele in the mouse model (image adapted from https://www.mousephenotype.org^49^). (**B**) Breeding statistics of breeding pairs carrying one wild-type and one knock-out allele (+/−) each (n = 19–45). (**C**) Peripheral blood analysis of hematocrit (HCT) (n = 13–19); (**D**) Hemoglobin concentration (HGB) (n = 13–19); (**E**) Red blood cell count (RBC) (n = 13–19); (**F**) Mean corpuscular volume (MCV) (n = 13–19); (**G**) Mean corpuscular hemoglobin (MCH) (n = 13–19); (**H**) Mean corpuscular hemoglobin concentration (MCHC) (n = 13–19) and (**I**) Red cell distribution width (RDW_SD) (n = 7) by utilization of a hematology analyzer. (**J**) Percentage of reticulocytes in total red blood cells as quantified by flow cytometry (n = 13). (**K**) Erythropoietin concentration in serum as detected by enzyme-linked immunosorbent assay (ELISA) (n = 9). Data displayed as average ± SEM, *P ≤ 0.05, ****P ≤ 0.0001.

To study how *Ypel4* affects erythropoiesis, we obtained a conventional knockout mouse model in which a targeted trap allele disrupts transcription of the protein-coding exons (Fig. 1A). The genotype was verified by PCR using primer combinations for allele-specific amplification (Supplementary Figure S1A). The *Ypel4*-null allele (–) was validated by RNA sequencing (Supplementary Figure S1B).

*Ypel4*-null (−/−) mice were born viable in Mendelian ratio, demonstrating that *Ypel4* is not essential for embryonic development **(**Fig. 1B, p = 0.74).

Peripheral blood (PB) from wild-type (+/+) and *Ypel4*-null mice was analyzed and blood values quantified in a hematology analyzer. *Ypel4*-null mice displayed a significant increase in hematocrit (HCT) (Fig. 1C) but not in hemoglobin concentration (HGB) or red blood cell count (RBC) (Fig. 1D,E). This was accompanied with a significant increase in mean corpuscular volume (MCV) (Fig. 1F), while mean corpuscular hemoglobin (MHC) (Fig. 1G) and mean corpuscular hemoglobin concentration (MCHC) (Fig. 1H) remained unaltered. Neither white blood cell count (WBC) (Supplementary Figure S1C) nor platelet count (Supplementary Figure S1D) was significantly changed.

To evaluate the characteristics of the observed macrocytosis, the RBC distribution width (RDW-SD) and reticulocyte count were measured using a hematology analyzer and flow cytometer, respectively. A trend towards relative anisocytosis, i.e. unequally sized RBCs, was observed in *Ypel4*-null mice (Fig. 1I), together with a slight but significant reticulocytosis (Fig. 1J).

To determine if the detected polycythemia, i.e. increase in HCT, and reticulocytosis had a primary or secondary cause, serum EPO concentration was measured using ELISA. Serum EPO concentration was significantly increased in *Ypel4*-null mice (Fig. 1K).

Taken together, our data demonstrates that the *Ypel4*-null mice disrupts transcription of the *Ypel4* gene, and that this disruption leads to a macrocytic secondary polycythemia.

### *Ypel4* is upregulated during terminal erythropoiesis but deficiency does not affect normal erythroid precursor functionality in bone marrow

**Figure 2:**
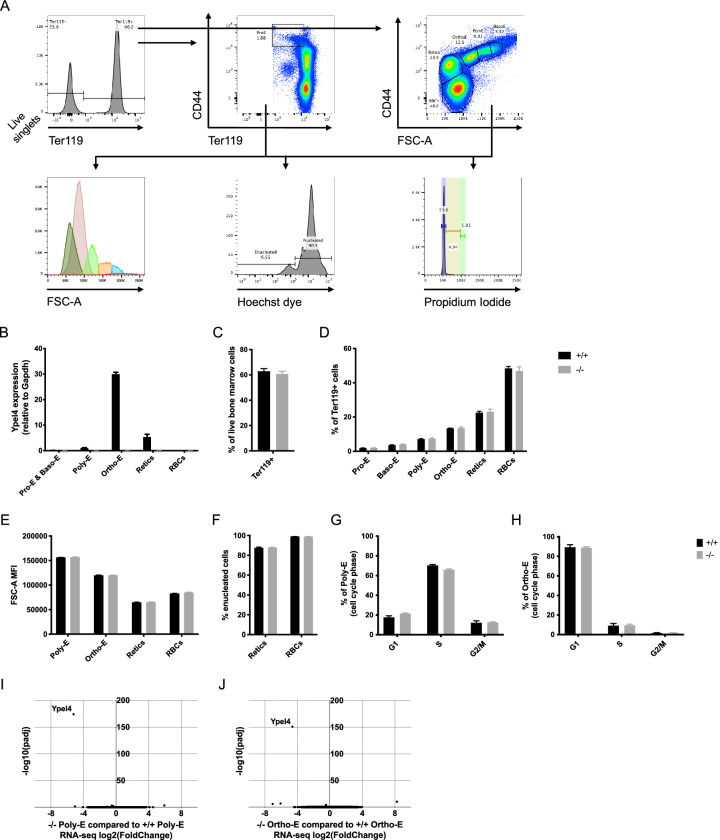
*Ypel4* is upregulated during terminal erythropoiesis but deficiency does not affect normal erythroid precursor functionality in bone marrow. (**A**) FACS gating strategy for bone marrow (BM) terminal erythroid cell populations in wild-type (+/+) and *Ypel4*-null (−/−) mice together with their forward scatter area (FSC-A) mean fluorescence intensity (MFI), Hoechst 33342 dye positivity and Propidium Iodide (PI) positivity after fixation. (**B**) *Ypel4* quantitative Polymerase Chain Reaction (qPCR) expression relative to *Gapdh* in sorted subpopulations (n = 3). (**C**) Total live erythroid cell (n = 4) and (**D**) subpopulation frequencies in BM (n = 4). (**E**) FSC-A MFI measurements as approximations of cell size in *Ypel4*-expressing subpopulations (n = 4). (**F**) Total enucleation efficiency as measured by lack of DNA staining using the cell-permeant Hoechst 33342 dye (n = 4). Cell cycle analysis by measurement of total DNA content (PI positivity) in the *Ypel4*-expressing (**G**) Poly-E (n = 3) and (**H**) Ortho-E (n = 3) populations after fixation. RNA-sequencing was conducted on sorted (**I**) Poly-E (n = 4) or (**J**) Ortho-E (n = 4) populations and differential gene expression presented using log2(fold change) and − log10(padj). Data displayed as average ± SEM. *Pro-E* proerythroblast, *Baso-E* basophilic erythroblast, *Poly-E* polychromatic erythroblast, *Ortho-E* orthochromatic erythroblast, *Retics* reticulocyte, *RBCs* red blood cells, *padj* adjusted p value.

To determine the cause of the peripheral blood phenotype, steady-state erythropoiesis in the bone marrow was examined using fluorescent activated cell sorting (FACS) and antibodies against the erythroid-specific marker Ter119. Inclusion of anti-CD44 antibodies allows for further subfractionation of the Ter119+ cells into proerythroblasts (Pro-E), basophilic erythroblasts (Baso-E), polychromatic erythroblasts (Poly-E), orthochromatic erythroblasts (Ortho-E), reticulocytes (Retics) and RBCs^23^. Within those populations, cell size, enucleation efficiency and cell cycle differences were analyzed (Fig. 2A).

Transcriptional analysis of the different Ter119+ subpopulations using FACS and qPCR revealed that *Ypel4* expression increased markedly by differentiation to peak in the Ortho-E population before enucleation. *Ypel4* mRNA was not detected in *Ypel4*-null populations (Fig. 2B).

In spite of being highly expressed during terminal erythropoiesis, total Ter119+ erythroid fraction in *Ypel4-*null bone marrow was not significantly different when quantified by flow cytometry (Fig. 2C). Neither could any significant differences in subpopulation frequencies within this fraction be detected (Fig. 2D). This was further confirmed with morphologic analysis of sorted erythroblast subpopulations stained with May-Grünwald-Giemsa (MGG) (Supplementary Figure S2A).

To analyze if *Ypel4*-deficiency affected normal erythroid precursor functionality, flow cytometry-based analyses to assess cell size, enucleation efficiency and cell cycle differences was carried out. Differences in cell size was analyzed by comparing the mean fluorescent intensity (MFI) of the forward scatter area (FSC-A) within the *Ypel4*-expressing Ter119+ subpopulations and RBCs. In spite of the observed macrocytosis in PB, no significant differences were detected between the compared groups (Fig. 2E). Neither could any differences be detected in the earlier erythroblast populations (Supplementary Figure S2B). Enucleation efficiency was analyzed by measuring positivity of a cell-permeant DNA dye (Hoechst 33342) in the reticulocyte and RBC populations. None of the compared groups displayed significant differences in enucleation rates (Fig. 2F). Cell cycle differences were analyzed in the *Ypel4*-expressing nucleated erythroblast populations using FACS and measurement of total DNA content using PI. No significant cell cycle differences could be detected between the groups (Fig. 2G,H). Neither could any difference be detected in earlier erythroblast populations (Supplementary Figure S2C).

In order to further analyze the effects on cellular processes, RNA-sequencing was performed on sorted Poly-E (Fig. 2I) and Ortho-E (Fig. 2J) populations from *Ypel4*+/+ and *Ypel4*−/− mice. The quality of the data was confirmed by a principal component analysis. Of note, corresponding wild-type and *Ypel4*-null samples did not cluster separate (Supplementary Figure S2D). In line with these findings, except for the expected changes in expression of *Ypel4* and the gene trapping splice acceptor *En2*, only three genes were significantly differentially expressed (P ≤ 0.05) in either population (Supplementary Table S1). Two of these genes were considered to be straight-forward false positive discoveries (Supplementary Figure S2E–G).

To analyze extra-medullary hematopoiesis, spleens were examined by weight which showed no significant difference in *Ypel4*-null mice (Supplementary Figure S2H). To investigate extra-medullary erythropoiesis in particular, flow cytometry and antibodies against Ter119 and CD71 were performed on spleens (Supplementary Figure S2I). Neither frequencies (Supplementary Figure S2J) nor total cell numbers (Supplementary Figure S2K) of early (CD71+Ter119+) or late (CD71−Ter119+) erythroid cells were significantly changed in *Ypel4*-null mice.

Taken together, in spite of high expression of *Ypel4* during late TED, disruption of *Ypel4* gene transcription does not significantly alter the late TED gene expression profile, erythroid precursor development, cell cycle or enucleation efficiency during steady-state erythropoiesis.

### *Ypel4*-null hematopoietic cells display reduced anemia-recovering capacity due to an increased clearance of circulating erythrocytes

**Figure 3:**
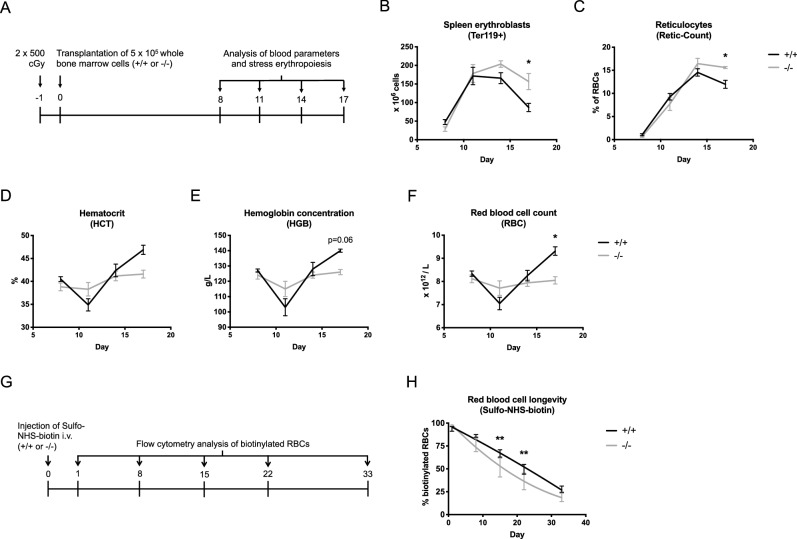
*Ypel4*-null hematopoietic cells display reduced anemia-recovering capacity due to an increased clearance of circulating erythrocytes. (**A**) Stress erythropoiesis was induced using lethal irradiation followed by transplantation of unfractionated wild-type (+/+) or *Ypel4*-null (−/−) bone marrow (BM) cells to +/+ recipients, followed by analysis of anemia recovery in spleen and peripheral blood (PB) at day 8, 11, 14 and 17. (**B**) Expansion of the Ter119+ erythroid fraction in spleen as quantified by cell counting and flow cytometry (n = 3). (**C**) Percentage of reticulocytes of total red blood cells as quantified by flow cytometry (n = 3). PB analysis of (**D**) hematocrit (HCT) (n = 3), (**E**) hemoglobin concentration (HGB) (n = 3) and (**F**) red blood cell (RBC) count (n = 3) by utilization of a hematology analyzer. (**G**) RBC longevity was analyzed by i.v. injection of Sulfo-NHS-biotin followed by flow cytometric quantification of biotinylated RBCs in PB at day 1, 8, 15, 22 and 33. (**H**) Percentage of biotinylated RBCs in PB over time with estimated clearance rates of wild-type (+/+) and *Ypel4*-null (−/−) RBCs (n = 5). Data displayed as average ± SEM, *P ≤ 0.05, **P ≤ 0.01, *i.v.* intravenous.

Due to the increased serum erythropoietin levels but lack of distinct phenotype in steady-state TED, we hypothesized that the kinetics of *Ypel4*-deficiency might be accentuated in a situation of induced anemia. Stress-erythropoiesis was therefore induced using a lethal dose of whole-body irradiation followed by transplantation of 5 × 10^5^ *Ypel4*+/+ or *Ypel4*−/− unfractionated BM cells (Ly5.2) to wild-type recipients (Ly5.1 + Ly5.2). Recovery was followed by analysis of PB, BM and spleen at day 8, 11, 14 and 17 after transplantation (Fig. 3A). Since spleen is the primary organ for stress-erythropoiesis in mice, we expected an early and robust response from spleen followed by a slower repopulation of the BM erythroid fraction^24^.

Using flow cytometry and cell counting, the Ter119+ erythroid fraction in spleen and BM was analyzed and quantified. As expected, a rapid expansion of the erythropoietic activity in spleen was observed between day 8 and day 11 in both groups of mice. Interestingly, mice transplanted with *Ypel4*−/− BM however displayed a prolonged expansion phase, generating more erythroid progenitors in the spleen compared to the control (Fig. 3B). As expected, the BM displayed a slower recovery rate than that seen in the spleen, with no significant difference between *Ypel4*+/+ and −/−-transplanted mice (Supplementary Figure S3A).

To analyze the output of reticulocytes, peripheral blood was stained with Retic-Count reagent and RNA/DNA-positive red blood cells detected by flow cytometry. In line with the increased stress-erythropoiesis observed in the spleen, the *Ypel4*−/−-transplanted mice kept producing more reticulocytes for a significantly longer time compared to *Ypel4*+/+-transplanted mice (Fig. 3C).

In order to trace the development and recovery of the anemia after irradiation, blood parameters were quantified using a hematology analyzer. In spite of the increased output of erythroid progenitors in spleen and reticulocytes in PB, mice transplanted with *Ypel4*−/− BM failed to recover their HCT (Fig. 3D), HGB (Fig. 3E) and RBC (Fig. 3F) over time compared to *Ypel4*+/+-transplanted mice, indicating an increased clearance of *Ypel4*−/− erythrocytes. The WBC count recovered at a similar rate (Supplementary Figure S3B), while the platelet count recovery rate was significantly decreased in the *Ypel4*−/− compared to the *Ypel4*+/+-transplanted mice (Supplementary Figure S3C).

To directly measure RBC longevity and rate of clearance, wild-type and *Ypel4*-null mice were injected with Sulfo-NHS-biotin that binds to and biotinylates RBCs. Mice were bled at day 1, 8, 15, 22 and 33 and the percentage of biotinylated RBCs was measured by flow cytometry (Fig. 3G). As expected, the wild-type mice had an estimated linear clearance rate of RBCs (Fig. 3H, black line), while RBCs in *Ypel4*-null mice were cleared at a significantly higher rate (Fig. 3H).

Taken together, *Ypel4*-null hematopoietic cells are equally able to expand the erythroid progenitor pool and form reticulocytes during stress-erythropoiesis as their wild-type counterparts. However, when they reach the circulation, *Ypel4*-null RBCs are cleared from the blood at a much higher rate compared to wild-type RBCs, indicating a lesion linked to the functionality of RBCs rather than erythroid precursors.

### Erythrocytes from mice deficient in *Ypel4* are ovalocytic, less deformable and have reduced Band 3 protein levels

**Figure 4:**
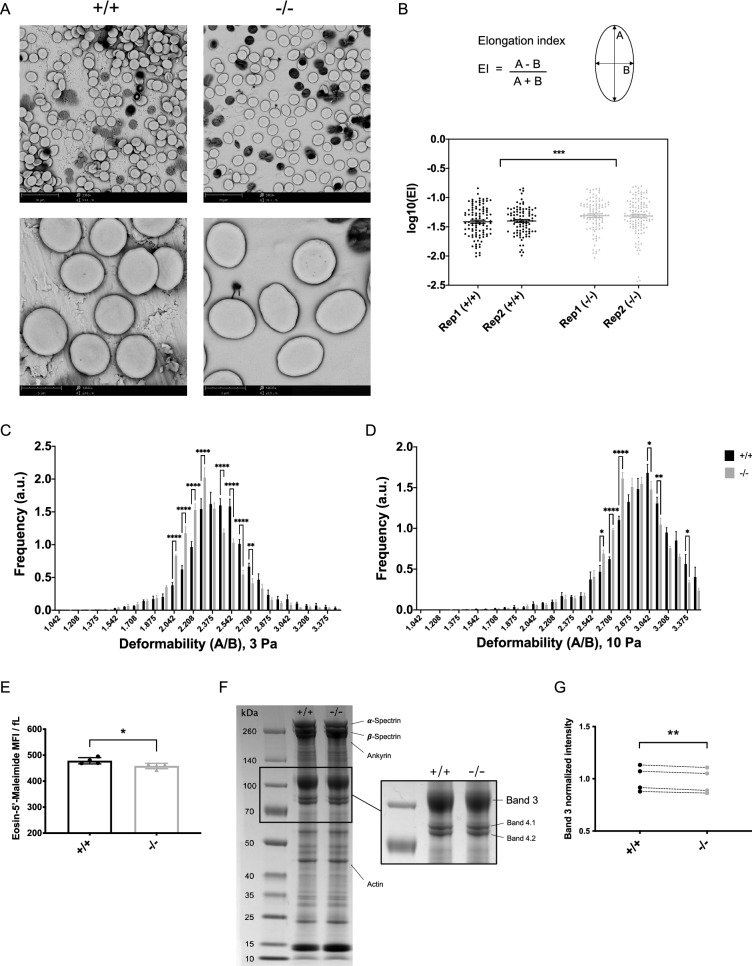
Erythrocytes from mice deficient in *Ypel4* are ovalocytic, less deformable and have reduced Band 3 protein levels. (**A**) Representative scanning electron micrographs of peripheral blood (PB) from wild-type (+/+) and *Ypel4*-null (−/−) mice. Micrographs taken at 3000 × and 13,000 × original magnification. (**B**) Isolated red blood cell (RBC) long and short axes were measured using ImageJ software and RBC elongation calculated using the elongation index (EI) formula *EI* = (*A* *−* *B)/*(*A* + *B*) (n = 2). (**C**,**D**) Deformability of RBCs was measured using an Automated Rheoscope and Cell Analyzer (ARCA) applying 3 Pa (**C**; n = 4) or 10 Pa (**D**; n = 4) shear stress. The graphs show the frequency of cells (y-axis) with a specific deformability index, this index is measured as the ratio between length over width (x-axis). (**E**) PB analysis of Eosin-5′-Maleimide mean fluorescence intensity (MFI) in RBCs measured by flow cytometry. MFI values are normalized to mean corpuscular volume (MCV), as determined by a hematology analyzer, in each sample for accurate density assessment of Band 3 protein levels in the RBC membranes (n = 4). (**F**) RBCs were hemoglobin-depleted using hypotonic lysis. Membrane protein concentrations were measured and equal amounts of protein solubilized and separated by sodium dodecyl sulphate (SDS)-polyacrylamide gel electrophoresis (PAGE). Gels were fixed in ethanol and stained with Coomassie Blue G-250 dye to visualize proteins. Representative images of one +/+ and one −/− sample is shown, first with all visible bands and then with only Bands 3, 4.1 and 4.2 visible after further separation to emphasize the visible differences in band intensities. (**G**) Band 3 intensities were analyzed and normalized by total protein normalization using ImageJ software. Data displayed normalized to average wild-type ratio and a paired Student’s *t* test was used to compare the means. Connecting lines depict age and gender matched +/+ and −/− samples (n = 4). All other data displayed as average ± SEM, *P ≤ 0.05, ** P ≤ 0.01, ***P ≤ 0.001, ****P ≤ 0.0001, *a.u*. arbitrary unit, *kDa* kilodalton.

Increased clearance of RBCs can be caused by different mechanisms^25^. Since YPEL family proteins have been observed to induce changes in cell morphology and the cytoskeleton^2,9^, we hypothesized that the increased clearance could be caused by a defect in the red cell membrane skeleton. To study the morphology, scanning electron microscopy was utilized to obtain high resolution micrographs of single RBC surface topographies. Examination of the micrographs revealed the presence of abnormally shaped erythrocytes in the *Ypel4*-null samples, identified as ovalocytes (Fig. 4A). Indeed, when calculating the elongation index for isolated RBCs in the samples, the majority of *Ypel4*-null RBCs displayed increased elongation compared to wild-type RBCs. There were no indications of RBC subpopulations (Fig. 4B).

The presence of abnormally elongated RBCs in the blood is indicative of both increased clearance of RBCs and reduced membrane deformability^26,27^. An Automated Rheoscope and Cell Analyzer (ARCA) was therefore utilized to evaluate the ability of individual RBCs to deform during shear stress. Deformability was evaluated at two different shear forces: 3 Pa and 10 Pa. At 3 Pa, differences between groups are often most prominent while at 10 Pa cells become more deformed. In accordance with the morphological observations, *Ypel4*-null RBCs were significantly less deformable than wild-type RBCs at 3 Pa and there were no detected subpopulations in the different groups (Fig. 4C). Similarly, a significant difference was detected even at 10 Pa when cells were much more uniformly deformed (Fig. 4D). The area of individual RBCs was also measured, demonstrating that *Ypel4*-null RBCs had a significantly larger area compared to wild-type RBCs (Supplementary Figure S4A), confirming the MCV results from the hematology analyzer.

The red cell membrane skeleton is built from cytoskeletal proteins anchored to Band 3 multiprotein complexes in the RBC membrane^28^. Flow cytometric analysis of Eosin-5′-maleimide (EMA) predominant binding to the Band 3 protein on intact RBCs is a routinely used first-line diagnostic screening test for hereditary red cell membrane disorders^29^. In disorders such as Hereditary Spherocytosis a clear reduction in EMA MFI is normally detected, while variants of Hereditary Elliptocytosis generally display a lower degree of reduction. The volume of RBCs affects the MFI, so the ratio between EMA MFI and MCV is a critical comparison^29,30^. An EMA binding test was therefore conducted together with hematology analyzer measurements of MCV. A slight but significant reduction in the EMA MFI to MCV ratio in *Ypel4*-null RBCs was detected (Fig. 4E).

The gold-standard technique to quantify relative membrane protein content is separation of solubilized and hemoglobin-depleted RBCs, so called RBC ghosts, by sodium dodecyl sulphate (SDS)-polyacrylamide gel electrophoresis (PAGE). The membrane proteins are visualized in the gel by Coomassie Brilliant Blue dye. Equal amounts of packed *Ypel4*-null and wild-type cells were loaded, and normalized intensities compared between matched samples (Fig. 4F, all samples shown in Supplementary Figure S4B). As indicated, Band 3 protein abundance in *Ypel4*-null RBC membranes was indeed slightly but significantly reduced compared to wild-type RBCs, confirming the results from the EMA binding test (Fig. 4G).

To elucidate if YPEL4 binds to the Band 3 protein directly, co-immunoprecipitation (Co-IP) was utilized to pull out YPEL4 protein complexes from primary erythroid cell lysates followed by quantitative liquid chromatography–mass spectrometry (LC–MS) analysis. Hematopoietic stem and progenitor (c-Kit+) cells from *Ypel4*−/− BM were transduced with either SFFV-FLAG-YPEL4 or SFFV-YPEL4-HA lentiviral overexpression vectors (Supplementary Figure S4C) and transplanted to lethally irradiated wild-type recipients. At day 16 after transplantation, recipients were sacrificed and total primary and erythroid cells in spleen recovered. Co-IPs cross-performed on cell lysates were analyzed by nano liquid chromatography–mass spectrometry (LC–MS) using a tandem mass tag (TMT) quantitative approach (Supplementary Figure S4D). Many proteins, including YPEL4, were detected as consistently enriched in the tagged compared to the control samples. The Band 3 protein however, even though detectable, was not amongst those (Supplementary Figure S4E, all detected proteins in Supplementary Table S2). These results provide support for the lack of physical interaction between YPEL4 and the Band 3 protein.

Taken together, erythrocytes from mice deficient in *Ypel4* are ovalocytic and less deformable than wild-type erythrocytes. This functional deficiency is associated with slightly reduced levels of Band 3 protein in the *Ypel4*-null RBC membranes, providing combined evidence for the presence of a mild red cell membrane disorder in the *Ypel4-*null mice. Lastly, strong indications are provided for an indirect effect of *Ypel4*-deficiency on Band 3 protein levels in RBC membranes.

## Discussion

The roles of the highly conserved YPEL family genes in mammals have for a long time been elusive. Most previous studies have been performed in vitro in cell lines. Here we describe in detail the role of *Ypel4* during erythroid development using a knockout mouse model, providing for the first time insights into the physiological processes governed by *Ypel4 *in vivo.

We demonstrated that *Ypel4*-deficiency leads to a secondary polycythemia in mice, with a slight increase in MCV that is accompanied by reticulocytosis. Since murine reticulocytes have around 45% more volume than erythrocytes^31^, the observed reticulocytosis likely contributes to the increase seen in MCV. However, the increase in reticulocyte numbers alone is not sufficient to explain the observed macrocytosis, since it would have required that the *Ypel4*−/− mice had more than 10% reticulocytes, compared to the 3.90 ± 0.18% observed. This conclusion is also supported by data from the ARCA experiment, showing a general shift in size of the whole RBC population. The secondary polycythemia displayed in the mice relates to a clinical condition, where an increase in RBC mass is seen together with increased serum EPO concentration. This condition is considered either physiologically appropriate, if there is decreased tissue oxygenation, or physiologically inappropriate, if there is abnormal overproduction of EPO^32^. In bone marrow transplantation and RBC longevity assays, we demonstrated an intrinsic RBC lesion and increased clearance of *Ypel4*-null erythrocytes. Mice transplanted with *Ypel4*-null cells also failed to recover platelet counts at the same rate as controls, an expected side-effect to the lingering anemia since megakaryocytopenia occurs naturally during situations of hypoxia and active erythropoiesis^33^. Our findings together provide evidence for a physiologically appropriate secondary polycythemia in the *Ypel4*-null mice.

YPEL4 and all other YPEL family proteins have previously been hypothesized to affect cell cycle regulation and progression due to their localization to mitosis-related structures^3–5^. In this study, even though we show that *Ypel4*-deficiency leads to macrocytosis, which can be due to cell cycle defects^34^, we do not detect a cell cycle defect in *Ypel4*-null erythroblasts. Our results, together with those from previous studies showing conflicting results regarding the effect of YPEL family proteins on cell cycle^5,35^, shows that this gene family might emerge as one with a much wider range of mechanisms than originally predicted.

*YPEL4* has also been shown to affect Elk-1 activity^4^. We, however, could not detect differences in Elk-1 target gene transcription in *Ypel4*-expressing bone marrow erythroblast populations. This negative finding is important for future studies aiming to investigate exactly how *YPEL4* affect Elk-1 activity. Of note, Elk-1 has been reported to acquire alternative functions during evolution^36^. It has also been uniquely reported to interact with neuronal microtubules^37^, relating to previous findings of YPEL family proteins to co-localize with microtubule-related structures^3–5^.

Despite the apparent lack of phenotype during TED, we could instead demonstrate that *Ypel4*-null RBCs were more elongated compared to normal RBCs with a resulting ovalocytic morphology. A discovery of changes in RBC morphology is a routine finding in the clinic when investigating a suspected red cell membrane disorder in a patient^26^. When further investigating the function of these altered RBCs, we found that they were cleared from the circulation at an increased rate and that they had reduced deformability. The Band 3 protein is fundamental in providing mechanical stability and deformability to the RBC, due to it being a major component in the macromolecular complexes serving as attachment points between the membrane and underlying cytoskeleton^38^. In various red cell membrane disorders, the Band 3 EMA MFI is affected negatively^39^. The results from the EMA binding test indicated a slight relative reduction of Band 3 protein levels in *Ypel4-*null RBC membranes. This reduction was confirmed by separation of solubilized RBC ghosts by SDS-PAGE. It remains an open question if the observed 2.2–4.4% reduction in Band 3 protein levels is sufficient to cause the pathologic alterations seen in *Ypel4*-null RBCs. Of note, it has been shown that the Band 3 EMA MFI is negatively correlated with osmotic fragility, implying that disease severity correlates with the degree of signal reduction^40^. It is therefore possible that a slight relative reduction in Band 3 protein levels could cause a mild red cell membrane disorder, especially considering the concurrent existence of abnormal RBC morphology in the *Ypel4*-null mice. A full investigation of the RBC membrane protein composition, including known Band 3 interacting proteins, was not conducted and is a delimitation of this study that should be taken into consideration.

We could not identify evidence of a physical interaction between YPEL4 and the Band 3 protein when performing two different Co-IPs, suggesting an indirect effect of YPEL4 on Band 3 protein levels. The remaining candidate binding partners could provide clues for future studies. Even though not verified as physical binding partners, the consistent enrichment of both alpha- and beta-tubulin in all Co-IP samples could be of special interest in the light of previous reports of YPEL family proteins to co-localize with microtubule-related structures^3–5^. Our findings further relate to previous studies showing that *Ypel1* induces changes in cell morphology, the cytoskeleton and cell adhesion machinery^2^, and *Ypel3* to be important for proper migration and morphology of perineural glia cells during neural development^9^. It is possible that redundancy between these highly similar YPEL proteins could attenuate the observed phenotype, although no changes in expression of other YPEL family genes could be detected in *Ypel4*-null erythroblasts.

In conclusion, we show that *Ypel4* is a highly specialized gene important for the integrity of the red cell membrane. We propose that through a putative indirect effect of *Ypel4*-deficiency, Band 3 protein levels are reduced in RBC membranes, likely contributing to the reduced membrane deformability, altered RBC morphology and increased clearance of RBCs in peripheral blood. These findings explain the observed secondary polycythemia in the *Ypel4*-null mice and provide evidence that the increase in hematocrit is a compensatory mechanism to secure proper tissue oxygenation. Further in-depth studies on the RBC membrane protein composition of the *Ypel4-*null mice might provide critical insights into the molecular function of *Ypel4* and the Yippee domain.

## Methods

### Mice

Mice were bred and maintained at the BMC animal facilities, Lund University, Sweden. The C57BL/6N-A^tm1Brd^ Ypel4^tm1a(EUCOMM)Wtsi^/WtsiIeg (Ly5.2) knockout mouse model was obtained from the European Mouse Mutant Archive.

Anemia was induced by subjecting age and gender matched adult recipient mice (C57BL/6xB6SJL; Ly5.1 + Ly5.2) to a single or split dose of lethal irradiation of 1 × 900 or 2 × 500 cGy, followed by bone marrow (BM) transplantation to rescue and trigger stress erythropoiesis. For analysis of recovery after anemia, 5 × 10^5^ unfractionated wild-type (+/+) or knockout (−/−) BM cells were transplanted intravenously (i.v.) by tail vein injection. The recipients were bled and sacrificed at either 8, 11, 14 or 17 days after transplantation for analysis of peripheral blood (PB), spleen and BM. For analysis of YPEL4 binding partners, 10^5^ transduced cKit + BM cells (−/−) were transplanted i.v. by tail vein injection. The recipients were sacrificed at day 16 after transplantation for recovery of primary erythroid cells in spleen.

For all types of blood analysis, PB was collected from the tail vein and analyzed according to described procedure.

For determination of blood parameters, PB was collected to EDTA coated Microvette tubes (Sarstedt, Nümbrecht, Germany) and analyzed on a KX-21N hematology analyzer (Sysmex, Kobe, Japan).

For determination of RBC longevity, an EZ-Link Sulfo-NHS-Biotin (Thermo Fisher Scientific, Waltham, MA, USA) solution was prepared in PBS, sterile-filtered and 3 mg administered i.v. by tail vein injection to each recipient at day 0. At day 1, 8, 15, 22 and 33 after injection, recipients were bled for analysis.

All procedures involving mice were approved by the Animal Ethics Committee of Malmö/Lund, Sweden and all experiments were performed in accordance with relevant guidelines and regulations. The reporting in the manuscript follows the recommendations in the ARRIVE guidelines.

### Enzyme-linked immunosorbent assay (ELISA)

The Mouse Erythropoietin Quantikine^®^ ELISA Kit (R&D Systems, Minneapolis, MN, USA) was used to detect serum erythropoietin (EPO) levels. Blood was collected to Eppendorf tubes without anticoagulant, and left at room temperature for one hour prior to being centrifuged at 2000*g*. Serum supernatant was collected and prepared according to manufacturer’s protocol. Samples were analyzed on SPECTROstar nano (BMG Labtech, Ortenberg, Germany). A standard curve was generated by four parameter logistic (4-PL) curve-fit to determine protein concentration of samples. Optical density was determined at 450 nm with a correction at 570 nm.

### Cell preparations and flow cytometry

BM and spleens were homogenized and filtered to single cell suspensions prior to cell culture, flow cytometry analysis or cell sorting (FACS).

For flow cytometry and FACS, the following conjugated antibodies against murine epitopes were used: CD44 (Clone IM7, APC) (BioLegend, San Diego, CA, USA), CD71 (Clone C2, PE) (BD Biosciences, San Jose, CA, USA), Ter119 (PE-Cy7) (eBioscience; Thermo Fisher Scientific), CD3 (Clone 145-2C11, PE-Cy5) (BioLegend), B220 (Clone RA3-6B2, PE-Cy5) (BioLegend) and Gr-1 (Clone RB6-8C5, PE-Cy5) (BioLegend). Fc receptor block (BD Biosciences) was used for sorting samples, dead cells were excluded using propidium iodide (PI) and enucleation was determined using Hoechst 33342 (Thermo Fisher Scientific).

Reticulocyte levels were determined by resuspending PB in BD Retic-Count (BD Biosciences) according to manufacturer’s protocol.

Biotinylated RBCs were detected with Streptavidin-conjugated PE-Cy7 (BioLegend).

Eosin-5′-Malemide (EMA) binding test was performed by resuspension of PB in EMA dye (Sigma-Aldrich, Saint Louis, MO, USA) according to manufacturer’s protocol.

Since erythroid precursors are very sensitive to fixation, cells from each of the nucleated erythroblast populations were sorted using FACS prior to fixation and staining for cell cycle analysis. Cell pellets were fixed with ice cold 70% ethanol in PBS while vortexing and incubated at − 20 °C overnight. The fixed cells were washed with PBS and stained with PI staining buffer [0.1% TritonX100 (Sigma-Aldrich), 0.1% Sodium citrate, 40 µg/ml PI, 500 µg/ml RNase A (Sigma-Aldrich)] for 30 min at 37 °C. PI intensity was analyzed individually in the sorted populations by flow cytometry analysis.

Flow cytometry analyses were performed on BD FACS Calibur or LSR II (BD Biosciences). FACS was performed on BD FACS Aria IIu or III (BD Biosciences). Results were analyzed with FlowJo 10 software (Tree Star, Ashland, OR, USA).

### RNA isolation and gene expression analysis

Total RNA was extracted from cells using RNeasy Kit (QIAGEN, Hilden, Germany) according to manufacturer’s protocol. Genomic DNA was removed using DNase I on-column digestion (QIAGEN).

For quantitative PCR (qPCR), complementary DNA from the isolated RNA was synthesized using the Superscript III Reverse Transcriptase (Invitrogen; Thermo Fisher Scientific) with random hexamers (Invitrogen; Thermo Fisher Scientific). Quantitation was performed on a 7900HT RT-PCR instrument (Applied Biosystems; Thermo Fisher Scientific) using the Mm.PT.58.9019795 (*Ypel4*) and Mm.PT.39a.1 (*Gapdh*) probes (Integrated DNA Technologies, Coralville, IA, USA).

For global gene expression, RNA concentration and integrity were measured using Agilent RNA Pico Kit on a Bioanalyzer Instrument (Agilent Genomics, Santa Clara, CA, USA). RNA sequencing libraries were prepared using TruSeq RNA Library Preparation Kit v2 (Illumina, San Diego, CA, USA) according to manufacturer’s standard protocol and the sequencing was performed using NextSeq 500/550 Mid Output v2 Kit, 150 cycle (Illumina). Fastq files were processed using the nf-core Nextflow pipeline for RNAseq *nf-core/rnaseq* v1.4.2^41,42^. Reads were mapped against GRCm38 reference genome. Differentially expressed genes were found using DESeq2^43^. Visual exploration and analysis of normalized sequencing data was performed using the Integrative Genomics Viewer software^44^.

### Scanning electron microscopy (SEM)

PB specimens were fixed with 2.5% glutaraldehyde in 150 mM sodium cacodylate pH 7.2 and adhered to a poly-l-lysine coated surface. They were washed with cacodylate buffer and dehydrated with an ascending ethanol series from 50% (v/v) to absolute ethanol. The specimens were then subjected to critical-point drying with carbon dioxide and absolute ethanol was used as an intermediate solvent. The tissue samples were mounted on aluminum holders, sputtered with 20 nm palladium/gold, and examined in a DELPHI correlative light and scanning electron microscope. Isolated RBC long and short axes were measured using ImageJ software^45^ and RBC elongation calculated using the elongation index (EI) formula *EI* = (*A* *−* *B*)/(*A* + *B*).

### Automated Rheoscope and Cell Analyzer (ARCA)

RBC deformability was measured with an ARCA and analyzed using automated cell framing software as described previously^46^. In short, morphological response to shear stress was measured at 3 Pa and 10 Pa. A minimum of 3000–4000 cells were measured and grouped in 30 bins according to increasing elongation or cell projection area (as a measure of membrane surface area). The elongation was calculated as the major cell radius divided by minor cell radius.

### Preparation of hemoglobin-depleted RBC ghosts

Equal volumes of packed cells were resuspended in ice cold PBS with added Halt Protease and Phosphatase Inhibitor Cocktail (Thermo Fisher Scientific), centrifuged cold at 2000*g* for 20 min and resuspended in 5 mM Na-Phosphate solution with added cOmplete Protease Inhibitor Cocktail (Sigma-Aldrich) to induce hypotonic lysis and generate RBC ghosts. The centrifugation and resuspension steps were repeated six times with the final centrifugation step prolonged with 10 min. The resulting hemoglobin-depleted RBC ghosts were resuspended in appropriate amount of Na-Phosphate solution with added protease inhibitors and membrane protein concentration determined using the Pierce 660 nm protein assay (Thermo Fisher Scientific) on a Nanodrop 1000 Spectrophotometer (Thermo Fisher Scientific).

### Sodium dodecyl sulphate (SDS)-polyacrylamide gel electrophoresis (PAGE) and staining

Equal amounts of protein were solubilized and boiled in 2 × Laemmli sample buffer (Bio-Rad Laboratories, Hercules, CA, USA) with 5% 2-Mercaptoethanol, then separated by sodium dodecyl sulphate (SDS)-polyacrylamide gel electrophoresis (PAGE). Gels were fixed in 30% ethanol, 2% v/v of 85% phosphoric acid overnight, washed 2 × 10 min in 2% (v/v) of 85% phosphoric acid and equilibrated for 30 min in 2% (v/v) of 85% phosphoric acid, 18% ethanol and 15% (w/v) ammonium sulphate. Equilibrated gels were stained with 0.2% Coomassie Blue G-250 dye (Sigma-Aldrich) for at least 30 min until proteins were visualized, analyzed in a ChemiDoc XRS+ with ImageLab software (Bio-Rad Laboratories) and band intensities quantified using ImageJ software^45^.

### Statistical analysis

All statistical significance, except where else stated, has been analyzed using either paired or non-paired Student’s *t* tests, Chi-square goodness of fit tests, nested *t* tests, one-way ANOVAs or 2-way ANOVAs followed by Tukey’s multiple comparisons test depending on experiments and groups compared. P values were adjusted for multiple comparisons where applicable. *P ≤ 0.05, **P ≤ 0.01, ***P ≤ 0.001, ****P ≤ 0.0001.

### Additional methods

For details about genotyping, staining procedures, molecular cloning and lentivirus production, cell culture, co-immunoprecipitation and proteomics, please refer to Supplementary Methods.

## Supplementary Information

Supplementary Information.

Supplementary Table S1.

Supplementary Table S2.

## Data Availability

The RNA-seq data discussed in this publication have been deposited in NCBI's Gene Expression Omnibus^47^ and are accessible through GEO Series accession number GSE171959. The mass spectrometry proteomics data have been deposited to the ProteomeXchange Consortium via the PRIDE^48^ partner repository with the dataset identifier PXD025242.
