# Supplementary material for: Yippee like 4 (Ypel4) is essential for normal mouse red blood cell membrane integrity
Source: Sci Rep. 2021 Aug 5;11:15898. doi: 10.1038/s41598-021-95291-1 (PMC8342551; doi:10.1038/s41598-021-95291-1)
Supplement: Supplementary file 1 — Supplementary Information. [file 41598_2021_95291_MOESM1_ESM.pdf]

Yippee like 4 (*Ypel4*) is essential for normal mouse red blood cell membrane integrity

Alexander Mattebo,[1] Taha Sen,[1] Maria Jassinskaja,[2] Kristýna Pimková,[2] Isabel Prieto González-Albo,[1] Abdul Ghani Alattar,[1] Ramprasad Ramakrishnan,[3] Stefan Lang,[2] Marcus Järås,[3] Jenny Hansson,[2] Shamit Soneji,[2] Sofie Singbrant,[1] Emile van den Akker,[4] and Johan Flygare*[1]

[1]Division of Molecular Medicine and Gene Therapy, Lund Stem Cell Center, Lund University, Sweden; [2]Division of Molecular Hematology, Lund Stem Cell Center, Lund University, Sweden; [3]Division of Clinical Genetics, Faculty of Medicine, Lund University, Sweden; and [4]Sanquin Research, Department of Hematopoiesis, Amsterdam, The Netherlands, and Landsteiner Laboratory, Academic University Medical Center, University of Amsterdam, Amsterdam, The Netherlands

*Correspondence: Johan Flygare, BMC A12, 221 84 Lund, Sweden; e-mail: johan.flygare@med.lu.se; phone number: +46 46 222 06 87; fax number: +46 46 222 05 68.

Supplementary Appendix

Supplementary Methods

DNA isolation and genotyping

Genomic DNA was prepared from mice ear marking samples and used as template for genotyping by PCR. A combination of one forward and two reverse primer oligonucleotides (5' to 3') were used to assess the genotype of mice progeny: Ypel4_222829_F GACCAGAGGACCAGATGCTAGG, Ypel4_222829_R GCCTTGAGTGCAACATCACC and CAS_R1_Term TCGTGGTATCGTTATGCGCC. The Ypel4_222829_F and Ypel4_222829_R primers amplifies a 406 basepair (bp) fragment with the wild-type (+) allele as template but fails to amplify a PCR fragment with the Ypel4$^{tm1a(EUCOMM)Wtsi}$ mutant (-) allele due to the inserted LacZ cassette. The Ypel4_222829_F and CAS_R1_Term primers amplifies a 292 bp fragment with the Ypel4$^{tm1a(EUCOMM)Wtsi}$ mutant allele but not with the wild-type allele, since the reverse primer anneals to a sequence in the inserted LacZ cassette. Gel electrophoresis was utilized for visualization of PCR products.

Cytospin and May-Grünwald-Giemsa staining

Cells were cytocentrifuged using Shandon Cytospin 3 (Block Scientific, Inc., NY, USA) at 350 rpm for 2 minutes. Giemsa solution was prepared by diluting Giemsa stock solution (Histolabs, Gothenburg, Sweden) to 5% in distilled water. Air-dried slides were stained in May-Grünwald solution (Merck, Darmstadt, Germany) for 5 minutes and then transferred to the Giemsa solution for 10 minutes. The slides were finally washed twice in distilled water and allowed to dry before examination under the microscope.

Molecular cloning and production of lentiviral vectors

The self-inactivating lentiviral vectors used in this study were derived from the SFFV-RPS19 vector.[1] *Ypel4* cDNA from murine fetal liver was inserted downstream of the spleen focus-forming virus (SFFV) promoter, either with FLAG (SFFV-FLAG-YPEL4) or HA (SFFV-YPEL4-HA) tag fused to the N-terminus or C-terminus of the protein coding sequence, respectively. Following the *Ypel4* cDNA, an internal

ribosomal entry site (IRES) and the protein coding sequence for EGFP was used as reporter of gene expression. Lentiviral vectors were produced by the Vector Unit at Lund University as described previously.[1]

Cell culture and lentiviral transductions

Singularized BM cell suspensions were enriched for cKit+ cells using CD117 MicroBeads and a magnetic-activated cell sorting (MACS) separation column (Miltenyi Biotech, Bergisch Gladbach, Germany) according to manufacturer's instructions. Purified cKit+ cells were resuspended in StemSpan serum-free expansion medium (SFEM) (Stem Cell Technologies, Vancouver, Canada) supplemented with 1% penicillin/streptomycin (P/S) (Cytiva, Marlborough, MA, USA), 20 ng/mL murine interleukin-3 (mIL-3) (PeproTech, Rocky Hill, NJ, USA), 20 ng/mL human thrombopoietin (hTPO) (PeproTech), 100 ng/mL murine stem cell factor (mSCF) (PeproTech), 2 U/mL human erythropoietin (EPO) (LEO Pharma, Ballerup, Denmark) and 100 nM Dexamethason (Sigma-Aldrich, Saint Louis, MO, USA).

Newly seeded cells were transduced at a multiplicity of infection (MOI) of 7.5 using 5 μg/mL polybrene transfecting agent (Merck Millipore, Burlington, MA, USA), incubated for 6 hours at 37°C and 5% CO_2 and transduced a second time with the same MOI before continued incubation overnight. The next day, samples were washed twice with PBS supplemented with 2% fetal bovine serum (FBS) (Thermo Fisher Scientific, Waltham, MA, USA) before transplantation.

Co-immunoprecipitation (Co-IP)

$4x10^7$ snap-frozen spleen cells were dissolved in ice-cold lysis buffer consisting of PBS, 0.5% Igepal CA-630 (Sigma-Aldrich) and added Halt Protease and Phosphatase Inhibitor Cocktail (Thermo Fisher Scientific). Cell remnants and nuclei were removed by centrifugation at 10 000g for 5 minutes and supernatant taken in equal volumes for Co-IP. Anti-HA magnetic beads (Thermo Fisher Scientific) and Anti-FLAG M2 magnetic beads (Sigma-Aldrich) were used for Co-IP according to manufacturer's instructions. The protein complexes were eluted from the beads by resuspension in 2x Laemmli sample buffer (Bio-Rad Laboratories, Hercules, CA, USA) with 5% 2-Mercaptoethanol (Sigma-Aldrich). Samples were boiled for 10 minutes at 95°C, then

separated by sodium dodecyl sulphate (SDS)-polyacrylamide gel electrophoresis (PAGE) for a short time until all samples were 1-2 cm in the gels. Gels were fixed and stained in 1:4:5 v/v of acetic acid: Milli-Q H_2O: methanol with 0.1% Coomassie Blue R-250 dye (Sigma-Aldrich) for 2 hours in room temperature (RT) before destaining in 1:3:6 v/v of acetic acid: methanol: Milli-Q H_2O overnight in 4°C. After destaining, each sample was cut out from the gel separately into 1x1 mm^2 cubes and transferred to new tubes, then centrifuged briefly before processing for proteomics analysis.

Proteomics sample preparation

In the presented study we combined in-gel digestion with chemical TMT labeling. The method was adapted from Schmidt et al.[2] with slight modifications. Gel pieces were washed with acetonitrile (ACN) and 200 mM triethylammonium bicarbonate TEAB/ACN (1:1 v/v) until they became colorless. Gel pieces were dried in a speed-vac and re-hydrated in 50 mM TEAB/10 mM dithiothreitol. Samples were incubated in a thermomixer for 50 minutes at 56°C to reduce disulfide bonds. Reduced thiols were blocked by addition of iodoacetamide (final concentration 22.5 mM) and incubation at RT for 30 minutes in the dark. Gel pieces were washed several times in 50 mM TEAB to remove remaining reagents. Washed pieces were dried and rehydrated in 25 mM TEAB. Trypsin was added at a final concentration of 0.02 μg/μl and proteins were digested overnight at 37°C. Tryptic peptides were extracted by washing gel-pieces with 1% formic acid/2% ACN (v/v) followed by 50% ACN in LC-MS grade water. Peptide concentration was estimated using NanoDrop. Supernatant containing tryptic peptides was dried completely. Extracted peptides were dissolved in 50 mM TEAB. TMT labeling using TMT6plex reagents was performed in accordance with manufacturer's protocol. Immediately before use, TMT label reagents were equilibrated to room temperature. Vials containing 0.8 mg of TMT label were dissolved in 41 μL of anhydrous ACN. Dissolved TMT labels were added to the dissolved peptides at the ratio recommended by the supplier. Samples were incubated for 1 hour at 37°C. Reaction was stopped by addition of 5% hydroxylamine to the samples. Samples representing biological replicate and their negative controls were combined, dried completely, re-dissolved in 0.1% TFA and

cleaned-up using C18 micro-spin columns. Desalted samples were dissolved in 4% ACN/0.1% formic acid and 1 μg was injected for nanoLC-MS.

Liquid Chromatography- Mass Spectrometry (LC-MS) analysis

MS analyses were carried out on an Orbitrap Fusion Tribrid MS system (Thermo Scientific) equipped with a Proxeon Easy-nLC 1000 (Thermo Fisher) on a 120-minute linear gradient separation followed by synchronous precursor selection MS3 (SPS-MS3) method. Each sample was injected twice. Injected peptides were trapped on an Acclaim PepMap C18 column (3 μm particle size, 75 μm inner diameter x 20 mm length, nanoViper fitting). After trapping, gradient elution of peptides was performed on an Acclaim PepMap RSLC C18 100 Å column (2 μm particle size, 75 μm inner diameter x 250 mm length, nanoViper fitting). The mobile phases for LC separation were 0.1% (v/v) formic acid in LC-MS grade water (solvent A) and 0.1% (v/v) formic acid in ACN (solvent B). Peptides were first loaded with a constant flow of solvent A at 9 μl/min onto the trapping column. Subsequently, peptides were eluted via the analytical column at a constant flow of 300 nl/min. During the elution step, the percentage of solvent B was increased in a linear fashion from 5% to 10% in 2 minutes, then increased to 25% in 85 minutes and finally to 60% in an additional 20 minutes. The peptides were introduced into the mass spectrometer via a stainless steel nano-bore emitter (150 μm OD x 30 μm ID; 40 mm length; Thermo Fisher Scientific) and a spray voltage of 2.0 kV was applied. The capillary temperature was set at 275°C.

The mass spectrometer was operated in data-dependent mode. First full MS scan was performed in the Orbitrap in the range of m/z 380 to 1580 and at resolution 120000 FWHM using automatic gain control (AGC) setting 4.0e5 and a maximum ion accumulation time of 50 ms. The top ten most intense ions selected in first MS scan were isolated for ion trap collision-induced dissociation MS2 (CID-MS2) at a precursor isolation window width of 0.7 m/z using AGC setting 1.5e4 and a maximum ion accumulation time of 50ms. The CID energy was set to 35%. Directly following each MS2 experiment, the top ten most intense fragment ions in an m/z range of 400-1200 were selected for high energy collisional-induced dissociation MS3 (HCD-MS3). The MS3 scan range was 100-500 m/z, the fragment ion isolation

width was set to 2 m/z, the AGC was 1.0e5 and the maximum ion time 120 ms. Normalized collision energy was set to 55%.

Proteomic data analysis

MS raw data were processed using Proteome Discoverer (version 2.2; Thermo Scientific). All raw files were processed together in a single run. Enzyme was set to trypsin and a maximum of two missed cleavages were allowed. Carbamidomethylation was set as a fixed modification; methionine oxidation and acetylation of protein amino-terminus were selected as variable modifications. The derived peak list was searched using the Sequest HT node against the Swissprot mouse database (version 2017.07.05; 25 170 protein entries) together with commonly observed contaminants and reversed sequences for all entries. Precursor and fragment mass tolerance were set to 10 ppm and 0.6 Da, respectively. Unique and razor peptides were used for quantification. The co-isolation threshold was set to 75. A 1% false discovery rate (FDR) was required at both the protein level and the peptide level. Contaminants and reverse hits were excluded. Protein quantification was evaluated based on corrected TMT reporter ion intensities.

References

1 Jaako, P. *et al*. Gene therapy cures the anemia and lethal bone marrow failure in a mouse model of RPS19-deficient Diamond-Blackfan anemia. *Haematologica* **99**, 1792-1798, doi:10.3324/haematol.2014.111195 (2014).

2 Schmidt, C. & Urlaub, H. iTRAQ-labeling of in-gel digested proteins for relative quantification. *Methods Mol Biol* **564**, 207-226, doi:10.1007/978-1-60761-157-8_12 (2009).

3 Boehm, J. S. *et al*. Integrative genomic approaches identify IKBKE as a breast cancer oncogene. *Cell* **129**, 1065-1079 (2007).

4 Robinson, J. T. *et al*. Integrative genomics viewer. *Nat Biotechnol* **29**, 24-26, doi:10.1038/nbt.1754 (2011).

Supplementary Table Legends

Supplementary Table S1. RNA-seq of poly- and orthochromatic bone marrow erythroblasts in wild-type and *Ypel4*-null mice. The spreadsheet contains lists of expressed genes in the different sequenced erythroblast populations as well as information about differentially expressed between wild-type and *Ypel4*-null samples found using DESeq2. Gene symbol, log2 Fold Change (*Ypel4*-/- compared to *Ypel4*+/+), p value, adjusted p value and -log10 (adjusted p value) are shown in the gene lists.

Supplementary Table S2. Proteins detected in quantitative LC-MS analysis after co-immunoprecipitation of YPEL4 tagged by FLAG and HA. The spreadsheet contains a list of all detected proteins in the proteomic data analysis together with comparable protein abundance measurements from all analyzed samples.

Supplementary Figure Legends

Supplementary Figure S1. *Ypel4* transcription is efficiently disrupted in *Ypel4*-null mice and affects red blood cell parameters only. (A) Representative separation of gel electrophoresis genotype Polymerase Chain Reaction products: a single 406 basepair (bp) band represents a wild-type (+/+) genotype, one 406 and one 292 bp band represents a heterozygous (+/-) genotype and a single 292 bp band represents a homozygous (-/-) genotype. (B) Visual exploration and analysis of the *Ypel4* gene disruption using RNA sequencing of orthochromatic erythroblasts and Integrative Genomics Viewer software[3] (n=4). Peripheral blood analysis of (C) white blood cell count (WBC) (n=13-19) and (D) platelet count (PLT) (n=13-19). Data displayed as average ± SEM.

Supplementary Figure S2. Erythroblast morphology and transcriptomes are unaffected in *Ypel4*-null mice. (A) Representative light microscopy images of May-Grünwald-Giemsa stained cytospins of sorted Ter119+ erythroblast subpopulations from wild-type (+/+) or *Ypel4*-null (-/-) bone marrow. Original magnification x1000. (B) Forward scatter area mean fluorescence intensity measurements as approximations of cell size in early Ter119+ subpopulations (n=4). (C) Cell cycle analysis by measurement of total DNA content (Propidium Iodide positivity) in early Ter119+ subpopulations after fixation. (D) Principal Component Analysis of RNA sequenced samples (n=4). Visualization of mapped RNA sequencing reads from *Ypel4*-expressing erythroblast populations to the (E, F) *Gm19426* and (G) *Lrrc8d* gene loci using Integrative Genomics Viewer software (n=4).[4] (H) Spleen weight as percentage of total body weight (n=5). (I) Flow cytometry gating strategy for analysis of extra-medullary erythropoiesis in *Ypel4*-null mouse spleen. (J) Frequencies and (K) total cell numbers of early (CD71+Ter119+) and late (CD71-Ter119+) erythroid cells (n=5). Data displayed as average ± SEM, Pro-E: proerythroblast, Baso-E: basophilic erythroblast, Poly-E: polychromatic erythroblast, Ortho-E: orthochromatic erythroblast.

Supplementary Figure S3. Ter119+ bone marrow and white blood cell count recover normally after transplantation of *Ypel4*-null hematopoietic cells. (A) Expansion of the Ter119+ erythroid niche in bone marrow (BM) after transplantation

of unfractionated wild-type (+/+) or *Ypel4*-null (-/-) BM cells to lethally irradiated mice at day 8, 11, 14 and 17, as quantified by cell counting and flow cytometry (n=3). Peripheral blood analysis of (B) white blood cell (WBC) and (C) platelet (PLT) count recovery after transplantation, by utilization of a hematology analyzer (n=3). Data displayed as average ± SEM, *P≤0.05.

Supplementary Figure S4. The Band 3 protein is not detected as a binding partner to YPEL4. (A) Red blood cells (RBC) were subjected to shear stress (10 Pa) using an Automated Rheoscope and Cell Analyzer with single cell resolution. Masking software (See Methods) was used to calculate the area (μM) of the deformed RBC (n=4). (B) RBCs were hemoglobin-depleted using hypotonic lysis. Membrane protein concentrations were measured and equal amounts of protein solubilized and separated by sodium dodecyl sulphate (SDS)-polyacrylamide gel electrophoresis (PAGE). Gels were fixed in ethanol and stained with Coomassie Blue G-250 dye to visualize proteins (n=4). (C) Plasmid maps of the SFFV-FLAG-YPEL4 and SFFV-YPEL4-HA overexpression vectors used for the co-immunoprecipitation (Co-IP) experiments, visualized using SnapGene® software (from Insightful Science; available at snapgene.com). (D) Stress erythropoiesis was induced using lethal irradiation followed by transplantation of 10^5 transduced c-Kit+ *Ypel4*-null (-/-) bone marrow (BM) cells to wild-type recipients. At day 16 after transplantation, recipients were sacrificed and primary erythroid cells in spleen recovered, Co-IPs cross-performed on cell lysates and tandem mass tag (TMT) chemical labeling performed to utilize the possibility of quantitative liquid chromatography- mass spectrometry (LC-MS) analysis through synchronous precursor selection (SPS)-MS3 method. (E) Enriched proteins in tagged compared to control Co-IP samples, filtered based on positive enrichment in all biological replicates. Mean imputation was utilized to replace missing values (n=3 for anti-FLAG, n=2 for anti-HA). Data displayed as average ± SEM, **P≤0.01, ***P≤0.001.

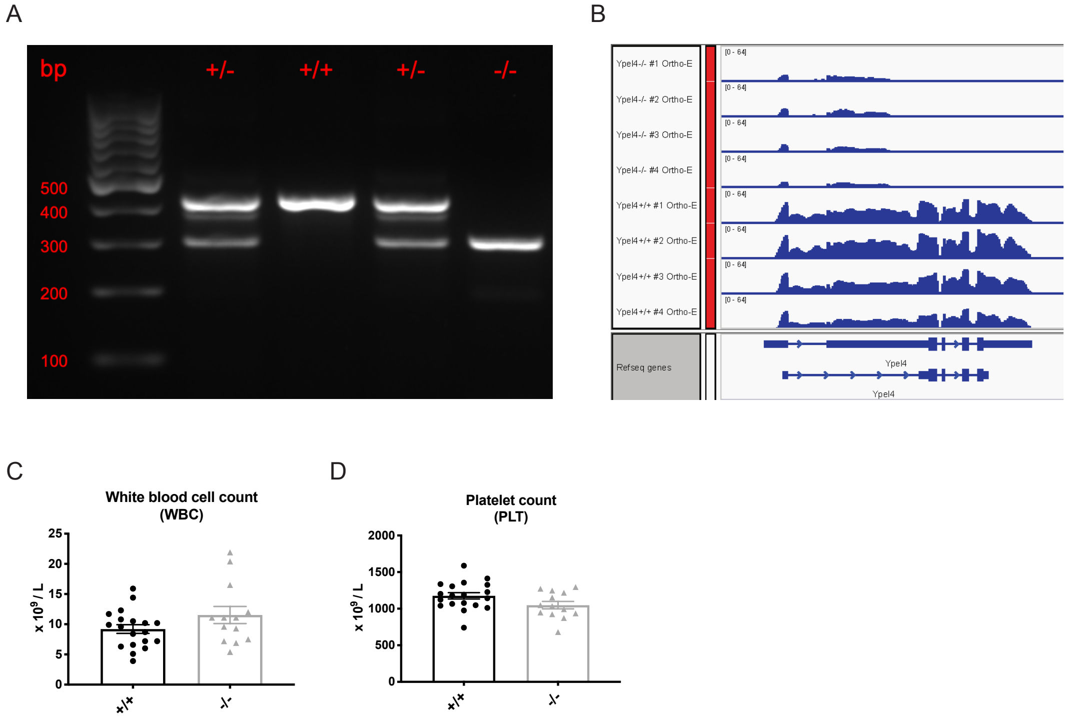

Supplementary Figure S1. ***Ypel4*** **transcription is efficiently disrupted in** ***Ypel4*****-null mice and affects red blood cell parameters only.**

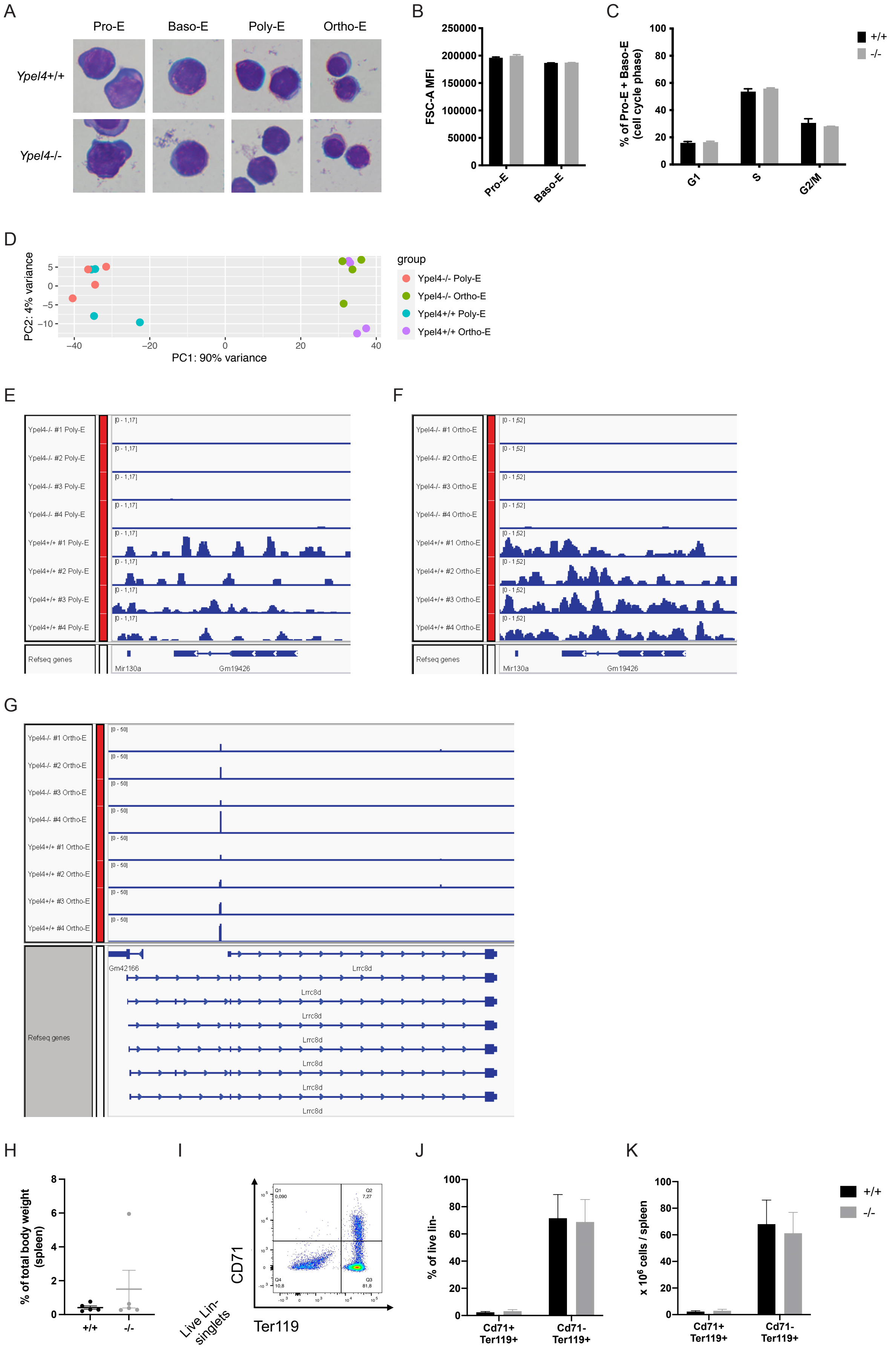

Supplementary Figure S2. Erythroblast morphology and transcriptomes are unaffected in *Ypel4*-null mice.

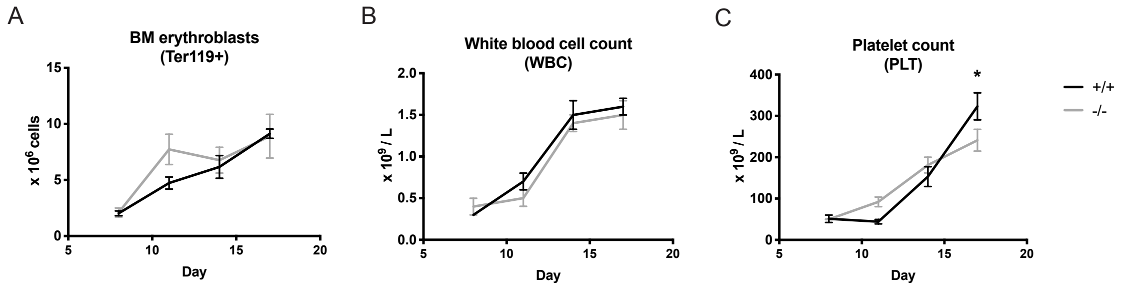

Supplementary Figure S3. Ter119+ bone marrow and white blood cell count recover normally after transplantation of *Ypel4*-null hematopoietic cells.

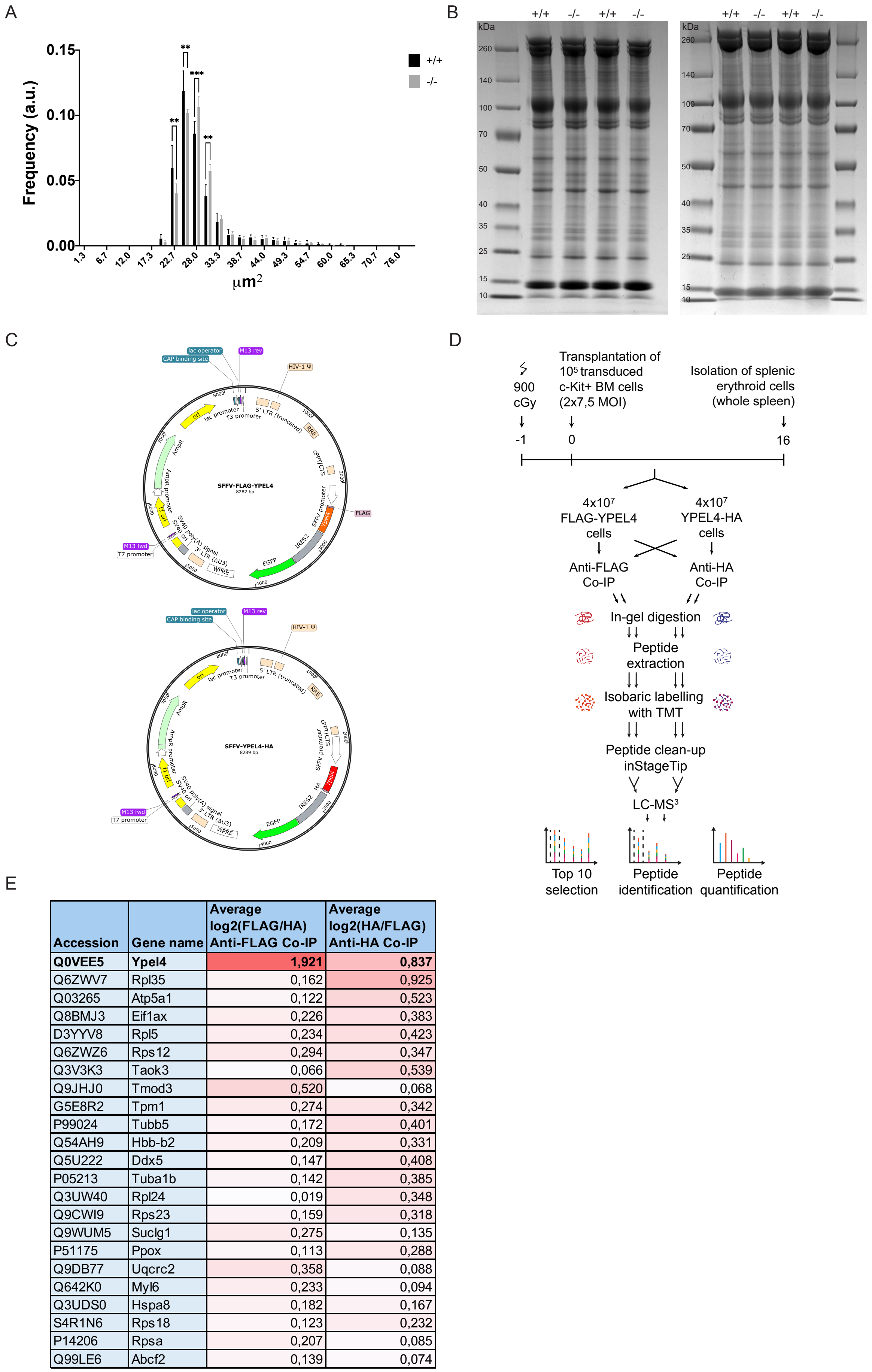

E

Accession	Gene name	Average log2(FLAG/HA) Anti-FLAG Co-IP	Average log2(HA/FLAG) Anti-HA Co-IP
**Q0VEE5**	**Ypel4**	**1,921**	**0,837**
Q6ZWV7	Rpl35	0,162	0,925
Q03265	Atp5a1	0,122	0,523
Q8BMJ3	Eif1ax	0,226	0,383
D3YYV8	Rpl5	0,234	0,423
Q6ZWZ6	Rps12	0,294	0,347
Q3V3K3	Taok3	0,066	0,539
Q9JHJ0	Tmod3	0,520	0,068
G5E8R2	Tpm1	0,274	0,342
P99024	Tubb5	0,172	0,401
Q54AH9	Hbb-b2	0,209	0,331
Q5U222	Ddx5	0,147	0,408
P05213	Tuba1b	0,142	0,385
Q3UW40	Rpl24	0,019	0,348
Q9CWI9	Rps23	0,159	0,318
Q9WUM5	Suclg1	0,275	0,135
P51175	Ppox	0,113	0,288
Q9DB77	Uqcrc2	0,358	0,088
Q642K0	Myl6	0,233	0,094
Q3UDS0	Hspa8	0,182	0,167
S4R1N6	Rps18	0,123	0,232
P14206	Rpsa	0,207	0,085
Q99LE6	Abcf2	0,139	0,074

Supplementary Figure S4. The Band 3 protein is not detected as a binding partner to YPEL4
